# Hepatoprotective Effect and Molecular Mechanisms of Hengshun Aromatic Vinegar on Non-Alcoholic Fatty Liver Disease

**DOI:** 10.3389/fphar.2020.585582

**Published:** 2020-12-04

**Authors:** Shenghu Zhu, Linshu Guan, Xuemei Tan, Guoquan Li, Changjie Sun, Meng Gao, Bao Zhang, Lina Xu

**Affiliations:** ^1^ Jiangsu Hengshun Vinegar Industry Co., Ltd., Zhenjiang, China; ^2^ College of Pharmacy, Dalian Medical University, Dalian, China; ^3^ Key Laboratory for Basic and Applied Research on Pharmacodynamic Substances of Traditional Chinese Medicine of Liaoning Province, Dalian Medical University, Dalian, China

**Keywords:** non-alcoholic fatty liver disease, aromatic vinegar, inflammation, lipid metabolism, silent information regulator of transcription 1

## Abstract

Aromatic vinegar with abundant bioactive components can be used as a food additive to assist the treatment of various diseases. However, its effect on non-alcoholic fatty liver disease (NAFLD) is still unknown. The purpose of this study was to investigate the mechanism of Hengshun aromatic vinegar in preventing NAFLD *in vivo* and *in vitro*. Aromatic vinegar treatment was applied to rats fed with a high-fat diet (HFD) and HepG2 cells challenged with palmitic acid (PA). Our results showed that aromatic vinegar markedly improved cell viabilities and attenuated cell damage *in vitro*. The levels of TC, TG, FFA, AST, ALT, and malondialdehyde (MDA) in HFD-induced rats were significantly decreased by aromatic vinegar. Mechanism investigation revealed that aromatic vinegar markedly up-regulated the level of silent information regulator of transcription 1 (Sirt1), and thereby inhibited inflammation of the pathway through down-regulating the expressions of high mobility group box 1, toll-likereceptor-4, nuclear transcription factor-κB, tumor necrosis factor receptor-associated factor-6, and inflammatory factors. Aromatic vinegar simultaneously increased the expression of farnesoid X receptor and suppressed expressions of lipogenesis related proteins, including fatty acid synthase, acetyl-CoA carboxylase-1, sterol regulatory element binding transcription factor 1, and stearoyl-CoA desaturase-1. These results were further validated by knockdown of Sirt1 using siRNAs silencing *in vitro*. In conclusion, Hengshun aromatic vinegar showed protective effects against NAFLD by enhancing the activity of SIRT1 and thereby inhibiting lipogenesis and inflammation pathways, which is expected to become a new assistant strategy for NAFLD therapy in the future.

## Introduction

Non-alcoholic fatty liver disease (NAFLD) is a clinicopathological syndrome characterized by excessive fatty deposition of hepatocytes, excluding alcohol and other well-defined liver damage factors. It is strongly associated with dyslipidemia, obesity, hypertension, and insulin resistance (Dowman et al., 2011). NAFLD encompasses a series of pathological changes ranging from simple hepatic lipid accumulation (steatosis) to non-alcoholic steatohepatitis (NASH) accompanied by inflammation and varying degrees of fibrosis (Soares e Silva et al., 2015). What is more serious is that NAFLD may even develop into eventual cirrhosis, hepatocellular carcinoma, and death related to liver disease (Farrell and Larter, 2006). In 2016, the global prevalence of NAFLD was about 25%, with the highest rates in South America and the Middle East and the lowest in Africa (Younossi et al., 2016). Statistics showed an upward trend in global morbidity, with an estimated increase of 3.6 million cases per year. It is estimated that the overall prevalence of NAFLD among adults will reach 33.5% by 2030 (Estes et al., 2018). Therefore, NAFLD has become an increasingly serious public health problem due to its rising incidence whether in developed or semi-developed countries.

Due to the occult nature of the disease, the specific pathogenesis of NAFLD is still unclear, and it is believed that NAFLD is affected by a variety of factors. At present, the “two-hit” hypothesis on the pathogenesis of NAFLD has been widely summarized and provides a profound theoretical basis for a series of subsequent studies (Basaranoglu et al., 2013). In the development of NAFLD, the “first hit” refers primarily to the abnormal accumulation of triglyceride (TG) in hepatocytes which has been confirmed to be associated with excessive lipid intake and insulin resistance (IR) (Bugianesi et al., 2010). Based on the first attack, the accumulation of lipids in hepatocytes destroys the oxidative capacity of mitochondria, leading to an increase in the number of reactive oxygen species involved in the re-reaction. Under the combined action of multiple cytokines, such mitochondrial oxidative stress ultimately leads to the “second hit” of NAFLD. It may trigger inflammation, fibrosis, and even hepatocytes apoptosis, ultimately causing end-stage liver disease (Rolo et al., 2012; Xia et al., 2019). Accordingly, we conclude that targeting the regulation of blood lipids and inflammatory responses will play an important role in the treatment of NAFLD.

At present, there is no effective treatment method for NAFLD, and commonly accepted treatment strategies mainly include weight loss, dietary changes, and physical exercise. With the widespread prevalence of NAFLD, people pay more and more attention to dietary changes as a preventive measure and therapeutic strategy for the disease. Studies have shown that Patchouli alcohol, a kind of medicinal food in Asian countries, has a protective effect against NAFLD by altering the metabolism (Wu et al., 2019). Other studies have indicated that tomato juice intake reduces triglycerides and cholesterol levels in the liver and serum, as well as the degree of hepatic steatosis (García-Alonso et al., 2017). Therefore, consumption of tomato juice or its extracts can improve the metabolic patterns of NAFLD induced by a high-fat diet (HFD). In addition, spinach can alleviate HFD-induced obesity, through increasing the number of Lactobacillus counts, lowering fasting blood glucose and total LDL-cholesterol, and preventing excessive cholesterol accumulation in the liver (Elvira-Torales et al., 2019).

Vinegar is a kind of fermented acidic liquid food, which is used as a condiment in cuisine. Previous studies have indicated that vinegar supplementation has a positive effect on the prevention of cardiovascular diseases, ulcerative colitis (UC), the recurrence of kidney stones, and that it can regulate blood glucose and blood pressure (Jing et al., 2015; Shen et al., 2016; Zhu et al., 2019). Moreover, dietary vinegar plays a certain role in reducing body weight, visceral and subcutaneous fat, and regulating lipid metabolism, it also has functional properties including antibacterial effects, antioxidation, control of blood glucose, anticancer properties and so on ( Kondo et al., 2009; Budak et al., 2014; Chen et al., 2016). A recent study demonstrated that synthetic acetic acid vinegar and Nipa vinegar had weight loss and anti-inflammatory effects on mice maintaining a HFD (Beh et al., 2017). However, the effect and molecular mechanism of aromatic vinegar on NAFLD remains unclear. Therefore, we conducted the following studies to investigate the hypolipidemic and hepatoprotective effects of Hengshun aromatic vinegar on NAFLD rats induced by a HFD and HepG2 cells induced by palmitic acid (PA) and elucidate the underlying molecular mechanism.

## Materials and Methods

### Chemicals and Materials

Hengshun aromatic vinegar was obtained from Jiangsu Hengshun Vinegar Industry Co., Ltd. (Zhenjiang, China). Dulbecco’s Modified Eagle’s medium (DMEM) and fetal bovine serum (FBS) were purchased from Gibco (California, United States). Oil Red O staining kit, 3-(4,5-Dimethylthiazol-2-yl)-2,5-diphenyltetrazolium bromide (MTT), PA, and simvastatin were supplied by Solarbio Science and Technology Co., Ltd. (Beijing, China). Total cholesterol (TC), TG, alanine aminotransferase (ALT), aspartate amino-transferase (AST), free fatty acids (FFA), and malondialdehyde (MDA) detection kits were obtained from Nanjing Jiancheng Institute of Biotechnology (Nanjing, China). The tissue protein extraction kit was purchased from KEYGEN Biotech (Nanjing, China). Enhanced bicinchoninic acid (BCA) protein assay kit was purchased from Beyotime Institute of Biotechnology (Jiangsu, China). TransZolTM, TransScript All-in-One First-Strand cDNA Synthesis SuperMix for qPCR, TransStart Top Green qPCR SuperMix, protein marker, EasySee Western blot kit were purchased from TransGen Biotech (Beijing, China). silent information regulator of transcription 1 (Sirt1) SiRNA (Sequences, sense: 5′-GGA​GAU​GAU​CAA​GAG​GCA​ATT-3′, antisense: 5′-UUG​CCU​CUU​GAU​CAU​CUC​CTT-3′) was designed and obtained from GenePharma Co., Ltd. (Shanghai, China). Lipofectamine2000 reagent was purchased from Thermo Fisher Scientific (Waltham, America).

### Cell Culture

HepG2 cells were purchased from the Shanghai Institute of Biochemistry and Cell Biology (Shanghai, China) and cultured in DMEM medium supplemented with 10% FBS in a humidified environment containing 5% CO_2_ and 95% O_2_ at 37°C.

### Cytotoxicity of Aromatic Vinegar

HepG2 cells were seeded into 96-well plates at a density of 5 × 10^4^ cells/well overnight, and treated with various concentrations of aromatic vinegar (0.125, 0.25, 0.5, 1, 1.5, 2, 3, and 4%) for 12, 24 and 36 h, respectively, and then the toxicity of the compound was assayed through the MTT method. A total of 10 µl MTT solution (5 mg/ml) was added to each well and incubated for 4 h, and then the formazan crystals were dissolved by 150 µl of DMSO. The absorbance of the specimen was quantified at 490 nm by a microplate reader (Thermo Fisher Scientific, MA, United States), and cell viabilities were calculated.

### Palmitic Acid Induced Injury in HepG2 Cells

A series of working dilutions of PA were prepared in a serum-free DMEM medium. HepG2 cells were seeded into 96-well plates at a density of 5 × 10^4^ cells/well overnight, and then treated with various concentrations (0.1, 0.2, 0.3, 0.4, 0.5, 0.6, 0.8 and 1.0 mmol/L) of PA for 12, 24 and 36 h. Cell viabilities were detected using the MTT method as described above and a suitable concentration of PA (0.3 mmol/L) was optimized.

HepG2 cells (5 × 10^4^ cells/well) were seeded into 96-well plates and incubated overnight. The cells in treatment groups were pretreated with aromatic vinegar at concentrations of 0.5, 1, and 1.5% for 24 h, and then treated with 0.3 mmol/L of PA for 24 h. Cells in the control group were cultured in serum-free DMEM medium under normal conditions for 48 h, and those for the model group were treated with 0.3 mmol/l of PA for 24 h. Finally, the viability of cell was determined with the MTT method as described above.

### Animals and Treatment

Seventy male Sprague-Dawley (SD) rats, weighing 180–220 g, were obtained from Liaoning Changsheng Biotechnology (SYXK (Liao) 2018-0007). The rats were kept under the controlled condition with constant temperature (23 ± 2°C), relative humidity (55% ± 5%), a 12 h light/dark cycle, and free access to water and food.

After 1 week of acclimatization, the rats were randomly divided into seven groups (n = 10), including the control group, the aromatic vinegar control group, the model group, the aromatic vinegar-treated groups (2.50, 1.25, 0.83 ml/kg aromatic vinegar), and the positive control group (4 mg/kg simvastatin). Normal saline was intragastrically administrated to the rats in the control and model group, and Hengshun aromatic vinegar and simvastatin were given to the treated animals once daily for 9 weeks. During this period, rats in the control and aromatic vinegar control group were fed a normal chow diet, while rats in other groups were fed a (HFD, consisting of 10% fat, 20% sucrose, 2.5% cholesterol, 1% cholate, 1% egg, 30% bean sprout and 35.5% chow diet) (Qin et al., 2018; Tang et al., 2018). Finally, the animals were sacrificed after an overnight fast, and serum samples were extracted from blood samples, centrifuged (3,500 rpm, 4°C) for 10 min and stored at −20°C until detection. The liver tissues were rapidly collected and stored at −80°C for other assays.

### Biochemical Assay

The levels of AST, ALT, TC, TG, and FFA in serum, and MDA in the liver tissue were determined using the detection kits based on the manufacturer’s instructions.

### H&E and Oil Red O Staining

In vivo experiments, liver tissues were fixed in 10% polyoxymethylene solution, processed by standard histological procedures, and ultimately embedded in paraffin wax. Then, the tissues were sectioned into 5-μm slices, which were stained with H&E. To evaluate lipid droplets in the liver, the frozen liver sections were stained with Oil Red O. In vitro experiments, HepG2 cells were treated with vinegar for 24 h and then the PA model was established for 24 h. After washing with PBS for 3 times, the cells were fixed with 10% paraformaldehyde solution for 30 min, and then stained with Oil Red O for 30 min finally washed with 60% isopropanol and PBS in sequence. Eventually, images of the stained sections and cells were obtained using a light microscope (NIKON, Tokyo, Japan).

### Immunofluorescence Assay

The paraffin sections of the liver were dewaxed twice with xylene for 15 min each time and rehydrated with different concentrations of alcohol (100, 90, 80, 70, and 60%) for 5 min. We then treated them with citrate buffer (pH = 6.0) in boiling water for 15 min for thermal repair. The deparaffinized liver tissue sections were incubated with rabbit anti-Sirt1, anti-farnesoid X receptor (FXR), or anti-high mobility group box 1 (HMGB1) (1:70, dilution) antibodies in a humidified box at 4°C overnight. After washing with PBS three times, the sections were incubated with a fluorescein-labeled secondary antibody for 1 h at 37°C. Eventually, the immunostained samples were imaged by fluorescence microscopy (Olympus, Tokyo, Japan).

### Real-Time PCR Assay

Total RNA samples were obtained from HepG2 cells and liver tissues using RNAiso Plus reagent following the manufacturer's protocol. After purity determination, cDNA was synthesized using a PrimeScript^®^ RT reagent Kit with a TC-512 PCR system (TECHNE, United Kingdom). The levels of mRNA were performed by using real-time PCR with SYBR^®^ PremixEx Taq TM II (Tli RNaseH Plus) in an ABI 7500 Real Time PCR System (Applied Biosystems, United States). The forward (F) and reverse (R) primers for the tested genes are listed in Table 1. Among the data from each sample, the Ct value of the target genes was normalized to that of GAPDH. The relative quantification of mRNAs was calculated using the 2^−△△Ct^ method.

**TABLE 1:** The primer sequences used for real-time PCR assay.

Genes	Forward primer (5′-3′)	Reverse primer (5′-3′)
Rats GAPDH	GGC​ACA​GTC​AAG​GCT​GAG​AAT​G	ATG​GTG​GTG​AAG​ACG​CCA​GTA
IL-1β	CCC​TGA​ACT​CAA​CTG​TGA​AAT​AGC​A	CCC​AAG​TCA​AGG​GCT​TGG​AA
IL-6	ATT​GTA​TGA​ACA​GCG​ATG​ATG​CAC	CCA​GGT​AGA​AAC​GGA​ACT​CCA​GA
TNF-α	TCA​GTT​CCA​TGG​CCC​AGA​C	GTT​GTC​TTT​GAG​ATC​CAT​GCC​ATT
Human GAPDH	GCA​CCG​TCA​AGG​CTG​AGA​AC	TGG​TGA​AGA​CGC​CAG​TGG​A
IL-1β	CTG​AGC​ACC​TTC​TTT​CCC​TTC​A	TGG​ACC​AGA​CAT​CAC​CAA​GCT
IL-6	TGG​CTG​AAA​AAG​ATG​GAT​GCT	TCT​GCA​CAG​CTC​TGG​CTT​GT
TNF-α	TGT​AGC​CCA​TGT​TGT​AGC​AAA​CC	GAG​GAC​CTG​GGA​GTA​GAT​GAG​GTA

TNF-α, tumor necrosis factor-α; IL-1β, interleukin-1β; IL-6, interleukin-6.

### Western Blotting Assay

Total protein samples from the liver tissues of rats and cells were extracted using a protein extraction kit and the protein content was determined by BCA Kit. Then, the proteins were loaded onto the SDS-PAGE gel (8–12%), separated electrophoretically, and transferred to PVDF membranes (Millipore, Massachusetts, United States). After being blocked with 5% dried skim milk for 3 h, the membranes were incubated with appropriate primary antibodies overnight at 4°C. The blots were then incubated with the goat anti-rabbit IgG-horseradish peroxidase-conjugated secondary antibody for 3 h. Protein expressions were detected by the enhanced chemiluminescence (ECL) method and the images were obtained by Bio-Spectrum Gel Imaging System (UVP, Upland, CA, United States). The intensity values of the relative protein levels were normalized with GAPDH as an internal control.

### Silent Information Regulator of Transcription 1 siRNA Treatment

HepG2 cells were cultured for approximately 24 h to 70–80% confluence in six-well plates. The cells were then transfected with Sirt1-targeted siRNA or empty vector plasmid (negative control, NC) using lipofectamine2000 reagent according to the manufacturer’s protocol. After transfection for 6 h, the medium was replaced with a complete medium. When the cells reached the appropriate density, they were exposed to aromatic vinegar (1.5%) for 24 h and treated with PA (0.3 mmol/l) for an additional 24 h. Then, the cells were collected for further experiments.

### Statistical Analysis

All data were expressed as the mean ± SD. Statistical analysis was performed using GraphPad Prism 5.0 software (San Diego, CA, United States). Significant differences among multiple groups were analyzed by a one-way ANOVA test followed by the Newman-Keuls test when comparing multiple independent groups. An unpaired t-test was carried out in the comparison two different groups. The results were considered to be statistically significant at *p* < 0.05. The data and statistical analysis were in accordance with standard recommendations for pharmacological experiment design and analysis.

## Results

### Aromatic Vinegar Attenuates Palmitic Acid-Induced Damage in HepG2 Cells

**FIGURE 1:**
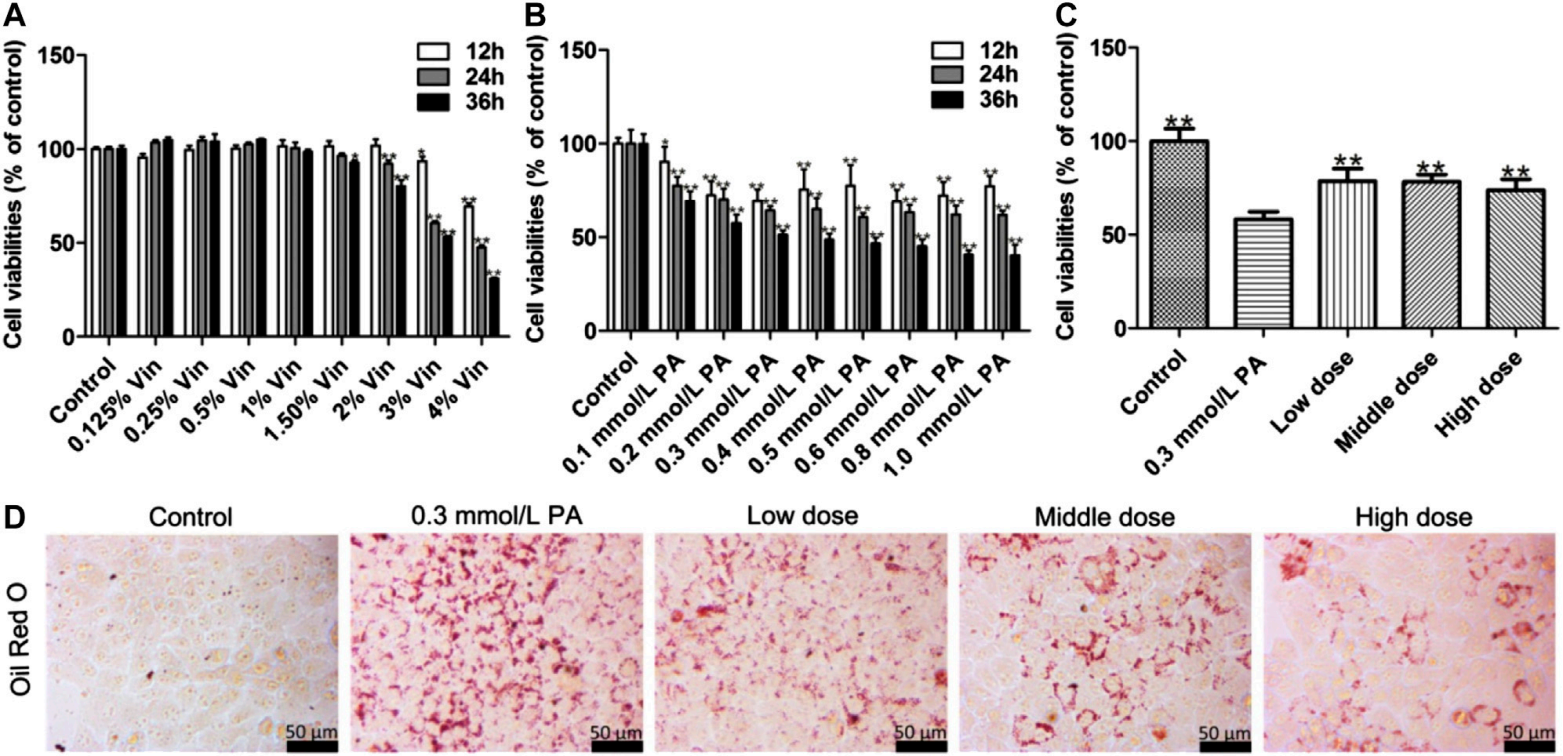
Protective effects of aromatic vinegar on palmitic acid (PA)-induced damage in HepG2 cells. **(A)** Cytotoxicity of aromatic vinegar (Vin) on HepG2 cells; **(B)** Effects of PA on cell viability; **(C)** Effects of aromatic vinegar attenuated on PA-induced cell damage; **(D)** Effects of aromatic vinegar on Oil Red O staining in HepG2 cells (×400 magnification; Scale bar = 50 µm). Values are expressed as the mean ± SD (n = 5). *p < 0.05, **p < 0.01 compared with model groups.

The results indicate that when the concentration of aromatic vinegar was higher than 1.5%, aromatic vinegar induced cell damage in HepG2 cells (Figure 1A). When the concentration of aromatic vinegar was less than 1.5%, the viability of HepG2 cells has no significant difference compared with the control group, indicating that aromatic vinegar was safe for HepG2 cells under the treatment conditions. Therefore, aromatic vinegar at concentrations of 0.5, 1, and 1.5% for 24 h were selected to protect HepG2 cells against PA-induced injury.

According to the results, the suitable concentration and treatment time of PA *in vitro* experiments were optimized. As shown in Figure 1B, compared with control groups, the viability rates of HepG2 cells were significantly decreased by 0.1–1.0 mmol/L PA treatment, which was decreased to 64.31% by 0.3 mmol/L of PA treatment for 24 h. Therefore, HepG2 cells were treated with 0.3 mmol/L PA for 24 h to establish the injury model.

In treatment groups, HepG2 cells were pretreated with aromatic vinegar at the concentrations of 0.5, 1, and 1.5% for 24 h, and then treated with 0.3 mmol/L of PA for 24 h. Subsequently, the results showed that aromatic vinegar pretreatment can significantly reverse the PA-induced cell damage and has an obvious protective effect on cells in a dose-dependent manner (Figure 1C).

As shown in Figure 1D, Oil Red O staining indicated that a large number of lipid droplet accumulations were observed in HepG2 cells after PA modeling, while aromatic vinegar treatment groups improved PA-induced lipid droplet accumulation compared with the model group.

### Effects of Aromatic Vinegar on Body Weight and Biochemical Indexes in Rats With Non-Alcoholic Fatty Liver Disease

**FIGURE 2:**
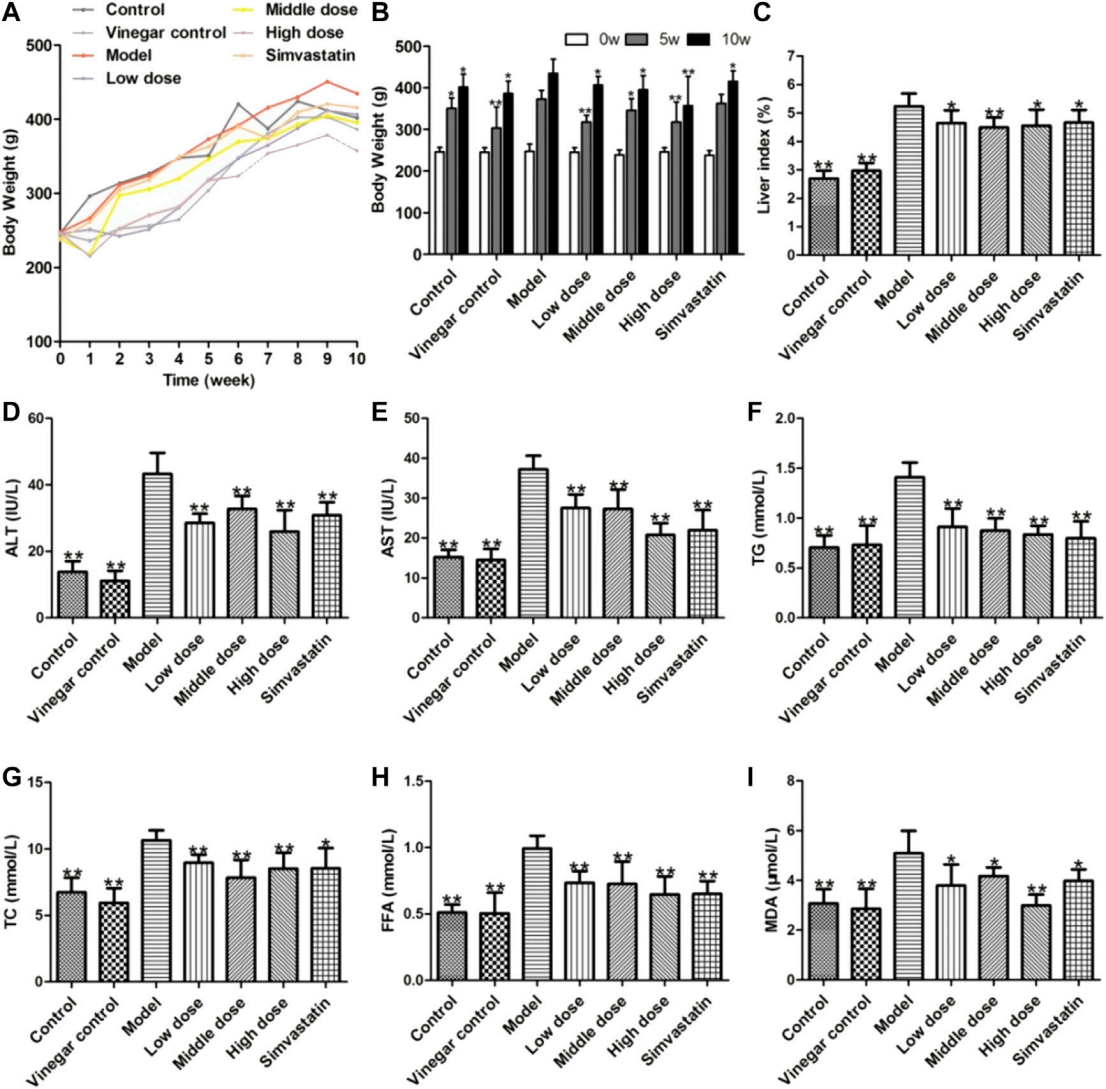
Effects of aromatic vinegar on biochemical indexes of rats. **(A,B)** Effect of aromatic vinegar on body weight **(C)** Effects of aromatic vinegar on the ratio of liver to body weight (%); **(D–I)** Effects of aromatic vinegar on the levels of alanine transaminase (ALT), aspartate transaminase (AST), TG, total cholesterol (TC), and free fatty acid (FFA) in serum and malondialdehyde (MDA) in liver tissue. Values are expressed as the mean ± SD (n = 8). *p < 0.05, **p < 0.01 compared with model groups.

The body weights of each group of rats were measured weekly. As shown in Figures 2A,B, the body weight of rats increased in each group after 10 weeks of feeding, while it was significantly improved in the aromatic vinegar and simvastatin administration groups compared with that of the model group. Similarly, the ratios of liver to body weight in aromatic vinegar groups and the simvastatin group were dramatically decreased compared with the model group (Figure 2C).

AST and ALT are specific aminotransferases in the liver, and their levels increase when the liver is damaged. As shown in **Figures 2D,E**, the levels of serum ALT and AST were significantly increased in the model group, while aromatic vinegar and simvastatin could markedly reduce these levels. Therefore, we concluded that feeding a HFD can cause liver damage in rats, while aromatic vinegar or simvastatin treatment can ameliorate liver damage. Similar results were shown in **Figures 2F,G,H**, the TG, TC, and FFA levels significantly increased in rats fed a HFD compared with the control group, while those were significantly reduced in aromatic vinegar groups. Moreover, as shown in Figure 2I, aromatic vinegar significantly reduced the MDA level in liver tissue compared with the model group. These data indicated that aromatic vinegar can ameliorate obesity and dyslipidaemia induced by a HFD, and significantly alleviate NAFLD via suppressing oxidative stress.

### Effect of Aromatic Vinegar on Liver Pathology

**FIGURE 3:**
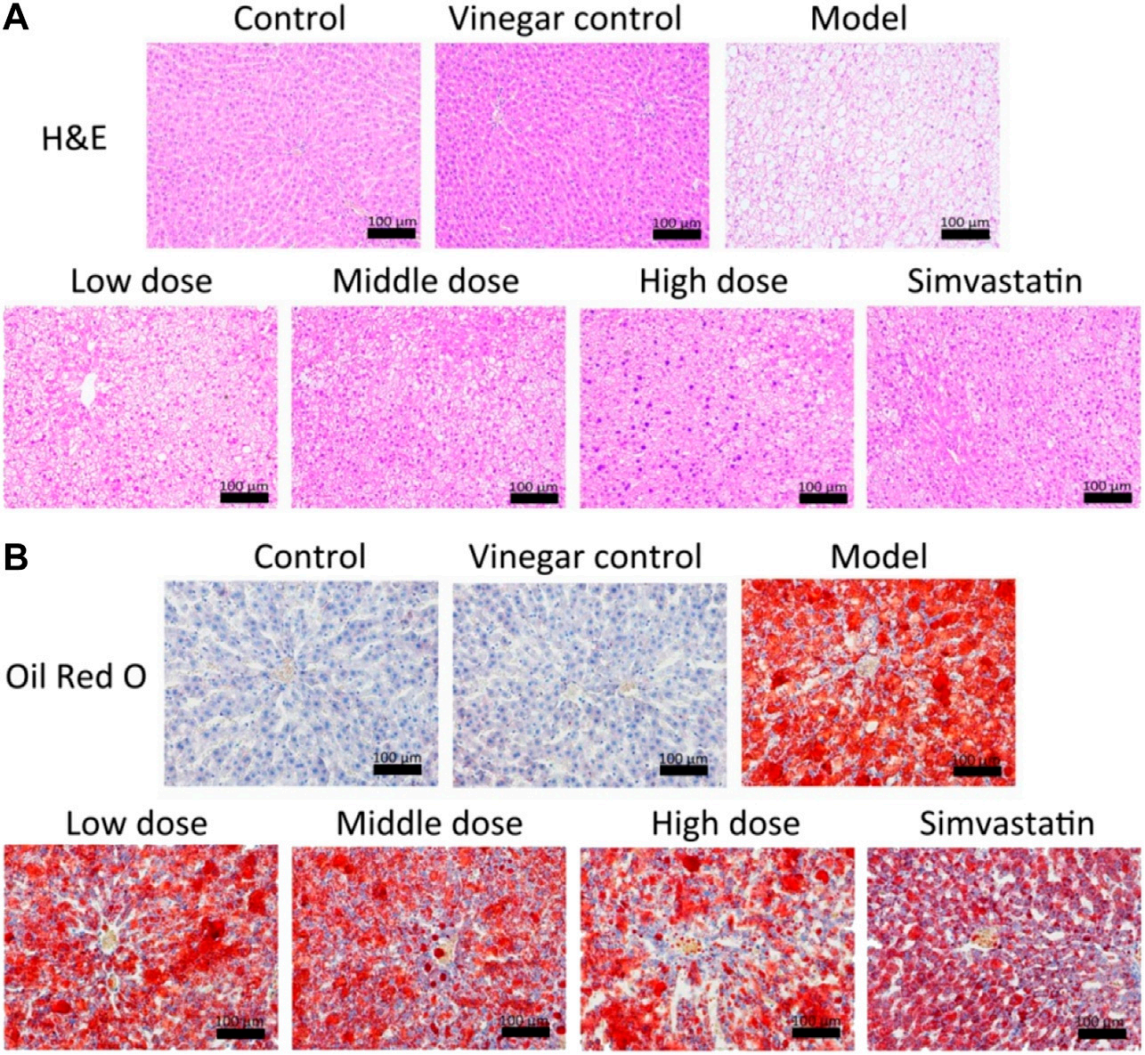
Effects of aromatic vinegar against non-alcoholic fatty liver disease in rats based on staining. **(A)** Effects of aromatic vinegar on HE staining images of liver tissues in rats (×200 magnification); **(B)** Effects of aromatic vinegar on Oil Red O staining images of liver tissues in rats (×200 magnification). Scale bar = 100 µm. Values are expressed as the mean ± SD. *p < 0.05, **p < 0.01 compared with model groups.

H&E staining showed that livers had an intact lobular architecture with a clear central vein and radiation line, and the cell cord is arranged neatly in the control group and vinegar control group. A large number of fat vacuoles and inflammatory cell infiltration appeared in the liver tissues of the model group, while aromatic vinegar and simvastatin administration significantly improved this condition (Figure 3A). In addition, compared with the control group, lipid droplets increased significantly in the HFD induced model, while administration groups significantly improved lipid accumulation in a dose-dependent way (Figure 3B). Oil Red O staining further showed that aromatic vinegar could inhibit excessive lipid accumulation in the liver. These data indicated that aromatic vinegar had a good effect on protecting the liver and reducing lipid.

### Aromatic Vinegar Activates Silent Information Regulator of Transcription 1 Pathway to Regulate Hepatic Lipid Synthesis and Inflammation *In Vitro* and *In Vivo*

**FIGURE 4:**
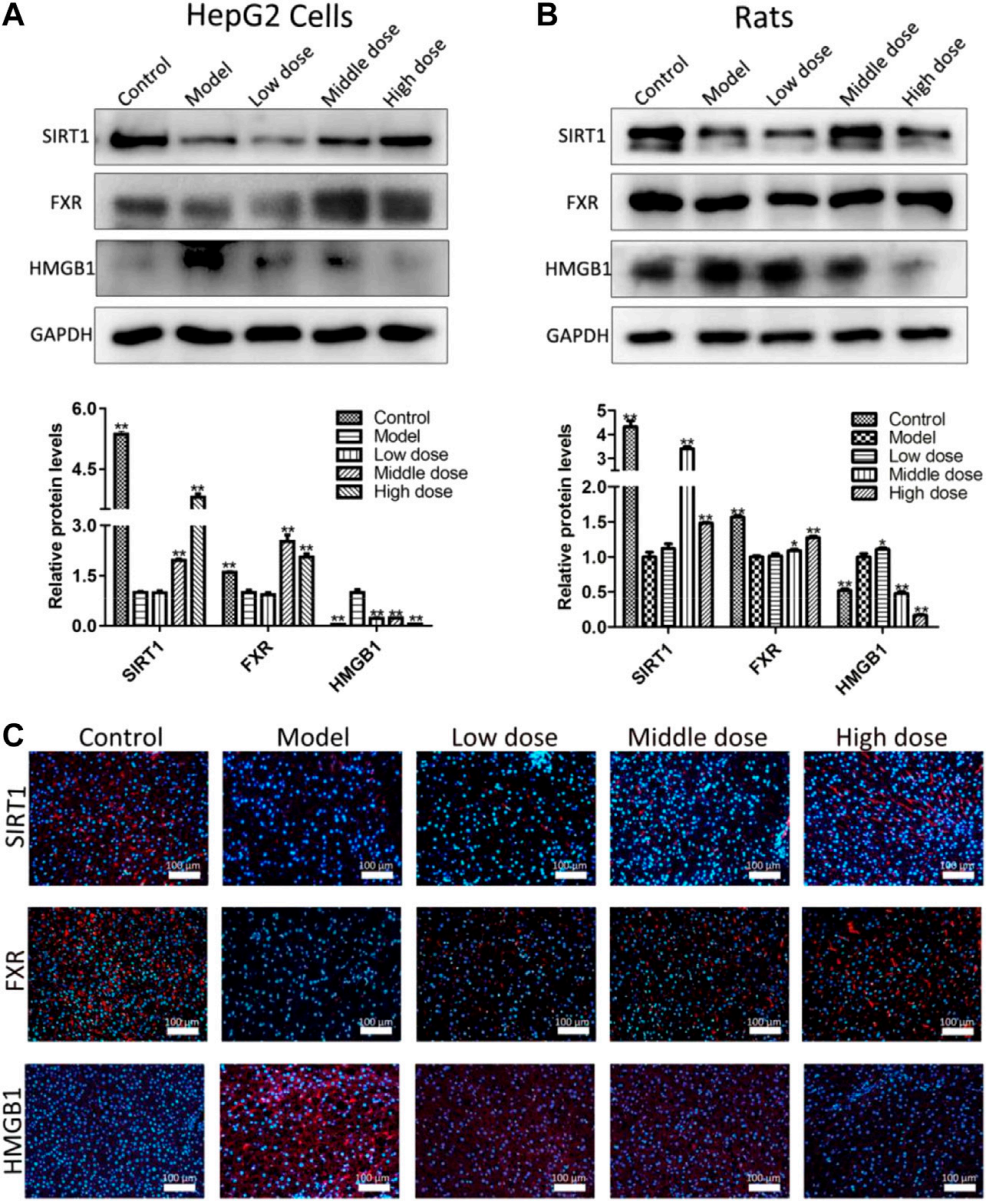
Aromatic vinegar activates the silent information regulator of transcription 1 pathway *in vitro* and *in vivo*. **(A)** Effects of aromatic vinegar on the expression levels of SIRT1, farnesoid X receptor (FXR) and high mobility group box 1 (HMGB1) *in vitro*; **(B)** Effects of aromatic vinegar on the expression levels of SIRT1, FXR and HMGB1 *in vivo*; **(C)** Effects of aromatic vinegar on SIRT1, FXR and HMGB1 levels based on immunofluorescence staining *in vivo* (×200 magnification). Scale bar = 100 µm. Values are expressed as the mean ± SD (n = 3). *p < 0.05, **p < 0.01 compared with model groups.

As shown in Figures 4A,B, the expression levels of SIRT1, FXR and HMGB1 in HepG2 cells and the liver tissues of rats were assessed by western blotting assay. The results indicated that the expression levels of SIRT1 and FXR were significantly down-regulated, and the level of HMGB1 was markedly up-regulated compared with control groups in vitro and in vivo. However, compared with model groups, aromatic vinegar significantly increased the expression levels of SIRT1 and FXR, and decreased the levels of HMGB1.

As shown in Figure 4C, the results indicate that the levels of SIRT1 and FXR-positive cells were substantially increased, and HMGB1-positive areas were obviously decreased in liver tissues by aromatic vinegar compared with the control groups based on immunofluorescence assays. These results indicated that aromatic vinegar activated the SIRT1 pathway, and then affected the expression levels of FXR and HMGB1.

### Aromatic Vinegar Ameliorates Lipid Metabolism *In Vitro* and *In Vivo*

**FIGURE 5:**
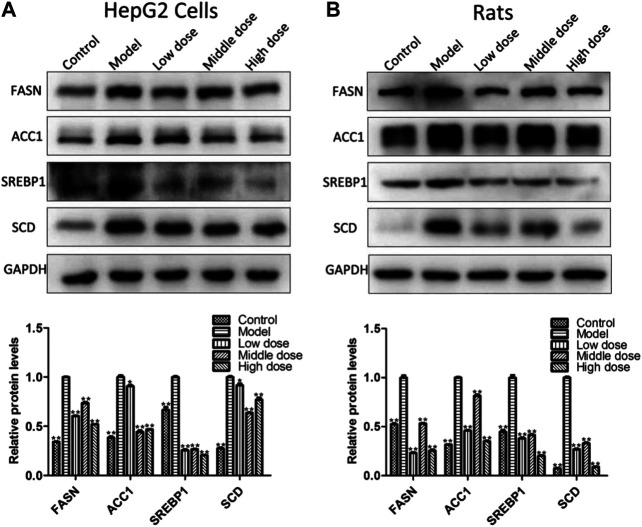
Effect of aromatic vinegar on the levels of some proteins related to lipid metabolism. **(A)** Effects of aromatic vinegar on the protein levels of fatty acid synthase (FASN), acetyl-CoA carboxylase-1 (ACC1), sterol regulatory element binding transcription factor 1 (SREBP1) and stearoyl-CoA desaturase-1 (SCD) *in vitro*; **(B)** Effects of aromatic vinegar on the protein levels of FASN, ACC1, SREBP1 and SCD *in vivo*. Values are expressed as the mean ± SD (n = 3). *p < 0.05, **p < 0.01 compared with model groups.

The results in Figure 5 show that aromatic vinegar markedly down-regulated the expression levels of proteins and genes associated with lipogenesis compared with the model groups *in vitro* and *in vivo*, including sterol regulatory element binding transcription factor 1 (SREBP1), fatty acid synthase (FASN), acetyl-CoA carboxylase-1 (ACC1) and stearoyl-CoA desaturase-1 (SCD1). The data indicated that aromatic vinegar could play an anti-NAFLD role by regulating lipid metabolism related proteins. 

### Aromatic Vinegar Attenuates Inflammation *In Vitro* and *In Vivo*

**FIGURE 6:**
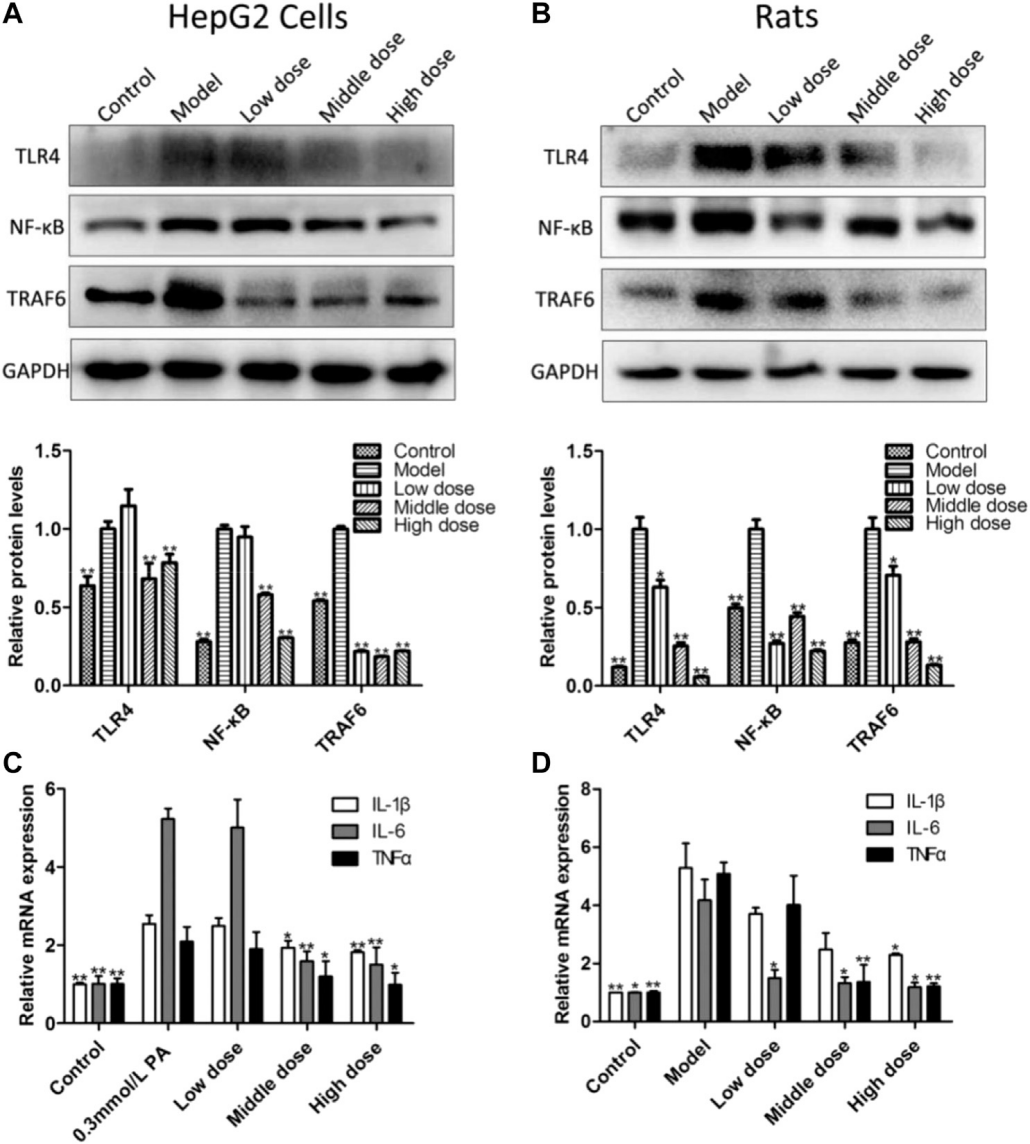
Effect of aromatic vinegar on the levels of some proteins related to inflammation and inflammatory mediators. **(A)** Effects of aromatic vinegar on the protein levels of toll-likereceptor-4 (TLR4), nuclear transcription factor-κB (NF-κB) and tumor necrosis factor receptor-associated factor-6 (TRAF6) *in vitro*; **(B)** Effects of aromatic vinegar on the protein levels of TLR4, NF-κB and TRAF6 *in vivo*; **(C)** Effects of aromatic vinegar on the mRNA levels of tumor necrosis factor-α (TNF-α), interleukin-1β (IL-1β) and interleukin-6 (IL-6) *in vitro*; **(D)** Effects of aromatic vinegar on the mRNA levels of TNF-α, IL-1β and IL-6 *in vivo*. Values are expressed as the mean ± SD (n = 3). *p < 0.05, **p < 0.01 compared with model groups.

As shown in Figures 6A,B, compared with control groups, the protein levels of toll-like receptor-4 (TLR4), nuclear transcription factor-κB (NF-κB) and tumor necrosis factor receptor-associated factor-6 (TRAF6) were all significantly increased, while the expression of which was significantly reversed by administrating aromatic vinegar *in vitro* and *in vivo*. As shown in **Figures 6C,D**, *in vitro* and *in vivo*, increased mRNA levels of tumor necrosis factor-α (TNF-α), interleukin-1β (IL-1β) and interleukin-6 (IL-6) were observed in the model groups, which were markedly decreased by aromatic vinegar. These results indicated that aromatic vinegar delayed NAFLD through ameliorating inflammation.

### Effect of Aromatic Vinegar on Lipid Metabolism and Inflammation After Transfecting Silent Information Regulator of Transcription 1 SiRNA *In Vitro*

**FIGURE 7:**
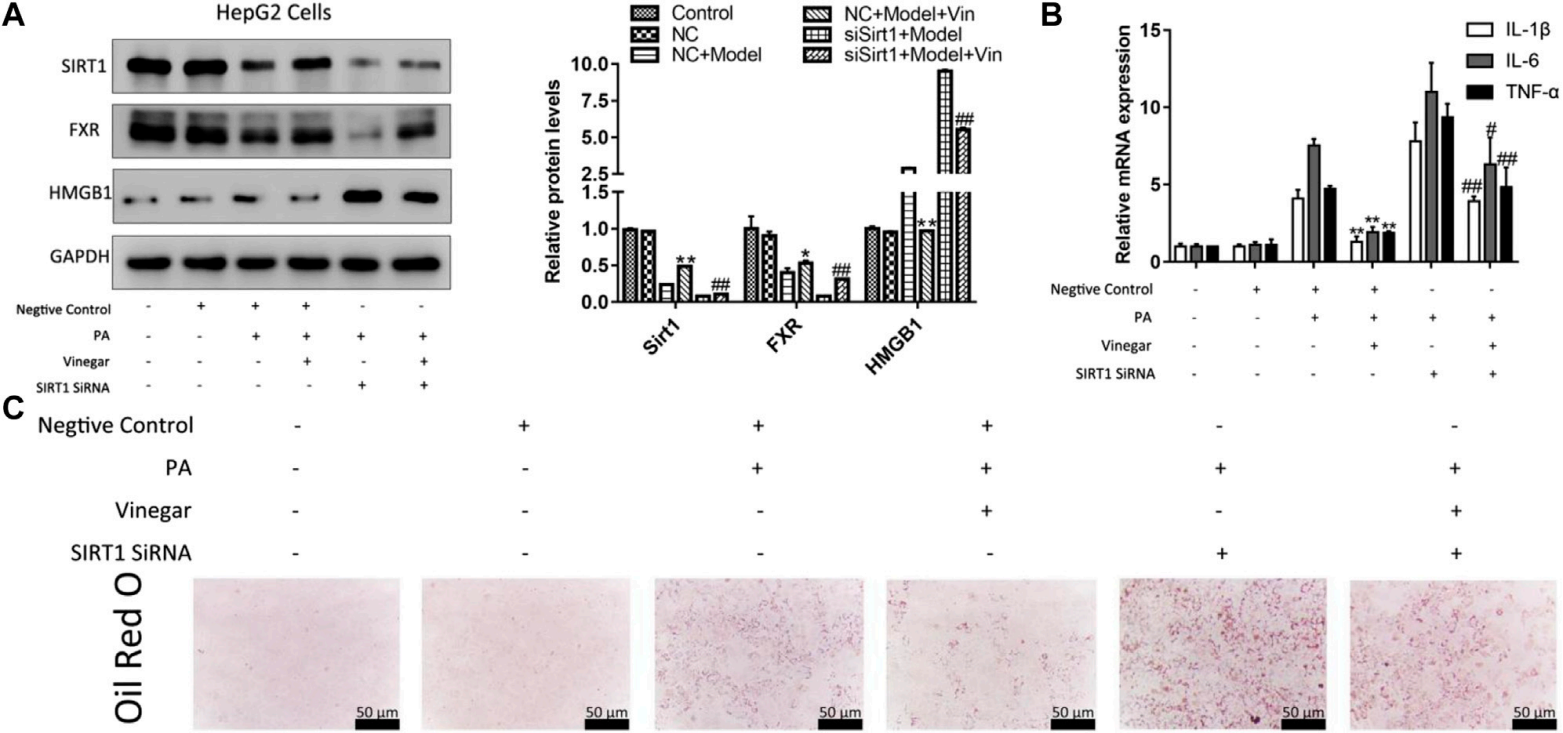
Effffect of aromatic vinegar on silent information regulator of transcription 1 (SIRT1)/FXR and SIRT1/high mobility group box 1 (HMGB1) pathway in HepG2 cells transfected with SIRT1 siRNA. **(A)** The protein levels of SIRT1, FXR and HMGB1; **(B)** The mRNA levels of interleukin-1β (IL-1β), interleukin-6 (IL-6) and TNF-α; **(C)** Oil Red O staining (×400 magnification; Scale bar = 50 µm). Values are expressed as the mean ± SD (n = 3). *p < 0.05, **p < 0.01 compared with model group transfected with negtive control siRNA (NC + Model); #*p* < 0.05, #*p* < 0.01 compared with model group transfected with SIRT1 siRNA (siSirt1 + Model).

To further determine the effect of aromatic vinegar on SIRT1 pathway, HepG2 cells were transfected with SIRT1 siRNA and pretreated with aromatic vinegar for 24 h before other tests. As shown in Figure 7A, transfecting SIRT1 siRNA aggravated PA-induced injury compared with the group transfected with negtive control siRNA. In addition, the protein levels of SIRT1 and FXR were markedly increased, and HMGB1 was markedly decreased in aromatic vinegar-treated groups after transfection. Obviously, SIRT1 siRNA reduced the protective effect of aromatic vinegar on PA-induced injury. What’s more, SIRT1 siRNA blocked the effects of aromatic vinegar on the mRNA levels of IL-1β, IL-6 and TNF-α (Figure 7B). Based on the Oil Red O staining shown in Figure 7C, aromatic vinegar treatment groups obviously improved PA-induced lipid droplet accumulation with or without SIRT1 siRNA. These results further strongly demonstrated that the regulatory effect of aromatic vinegar on lipid metabolism and inflammation was achieved through regulation of the SIRT1/FXR and SIRT1/HMGB1 signal pathways.

## Discussion

NAFLD is a type of acquired metabolic stress liver injury, which is closely related to insulin resistance and genetic susceptibility (Cheung and Sanyal, 2010). With the epidemic trend of obesity caused by unhealthy diet, the prevalence of NAFLD are rising worldwide, and NAFLD has become the main cause of chronic liver disease in developed countries (Safari and Gérard, 2019; Stefan et al., 2019). NAFLD is a major global health issue that deserves close attention. The pathogenesis of NAFLD is a complex process, in which the occurrence of hepatic lipotoxicity may further lead to mitochondrial dysfunction, thereby activating inflammatory responses (Zhang et al., 2018). The liver provides a central store for excessive lipid accumulation (Chung et al., 2017). NAFLD is characterized primarily by fatty deposition of hepatocytes, which accounts for more than 5% of the liver weight (Gong et al., 2017). As is known to all, Hengshun aromatic vinegar is one of the most famous traditional fermented vinegars in China, and is generally produced from glutinous rice, wheat bran, and rice hulls through a unique solid layered fermentation process (Xu et al., 2011a). It has the characteristics of “color, fragrance, acid, alcohol, thick,” and has a strong taste that is sour and not astringent, fragrant and slightly sweet, colory and strong taste. Although there is no accurate formulation for aromatic vinegar, it has a hundred years of mature, reliable production technology and quality control. What’s more, Chinese literature has found that Hengshun aromatic vinegar contains 88.09% water and volatile matter and 11.91% solid matter (w/v), and is rich in organic acids, sugar, protein, amino acids and other aromatic ingredients, which largely affect its taste characteristics and organoleptic quality (Zhao et al., 2018). The effective components in it are relatively abundant and stable through the fingerprint analysis and amino acid analysis. During the brewing process, aromatic vinegar produces many bioactive compounds, such as organic acids, amino acids and phenolic compounds, which play important roles in antioxidant activities (Zhang et al., 2019). In addition, polyphenols, flavonoids, and melanoidins were found in Hengshun aromatic vinegar, which play crucial roles in diseases prevention and contribute benefits to human health (Zhao et al., 2018; Duan et al., 2019). Studies have also showed that the alkylpyrazine named ligustrazine was found as a bioactive compound in Hengshun aromatic vinegar, which has the health effects such as reducing blood pressure, promoting blood circulation and removing stasis, improving coronary heart disease, thrombolytic therapy and liver protection (Xu et al., 2011b; Chen et al., 2019). In the present study, PA-induced HepG2 cells and HFD-induced SD rats were used as NAFLD models *in vitro* and *in vivo* to investigate the pharmacodynamic actions and molecular mechanism of Hengshun aromatic vinegar. Aromatic vinegar showed significant effects against NAFLD due to the evidences with the decreased levels of AST, ALT, TC, TG, FFA and MDA, and the alleviation of histopathological changes.

SIRT1, a nonamide adenine dinucleotide (NAD^+^)-dependent protein deacetylase, acts as an important modulator of metabolic pathways and plays a critical role in the pathophysiology of many metabolic diseases. It’s a member of silent information regulator 2 family, which are a group of proteins with either histone deacetylase or mono-ADP-ribosyltransferase activity (Colak et al., 2011). In recent years, studies have shown that SIRT1 with an extensive spectrum of biological functions plays an important role in the regulation related to metabolism, cell survival and apoptosis (Silva and Wahlestedt, 2010). In addition, it has been found that the pathophysiological mechanism of NAFLD may be affected by the activation of SIRT1 (Colak et al., 2014). Studies have shown that dioscin regulated lipid metabolism through SIRT1/AMPK signal pathway and significantly prevented NAFLD (Yao et al., 2018). Similarly, it has also been reported that celastrol ameliorated NAFLD by SIRT1 pathway, which had an important role in improving liver metabolic injury induced by HFD (Zhang et al., 2016). In this study, we did find the result that the expression of SIRT1 was down-regulated in model group, which was consistent with SIRT1 expression in other studies. Moreover, our SIRT1 siRNA experimental results proved that the main target of aromatic vinegar was SIRT1. It attenuated NAFLD by activating the SIRT1 pathway to regulate liver lipid metabolism and inflammation *in vitro* and *in vivo*.

**FIGURE 8:**
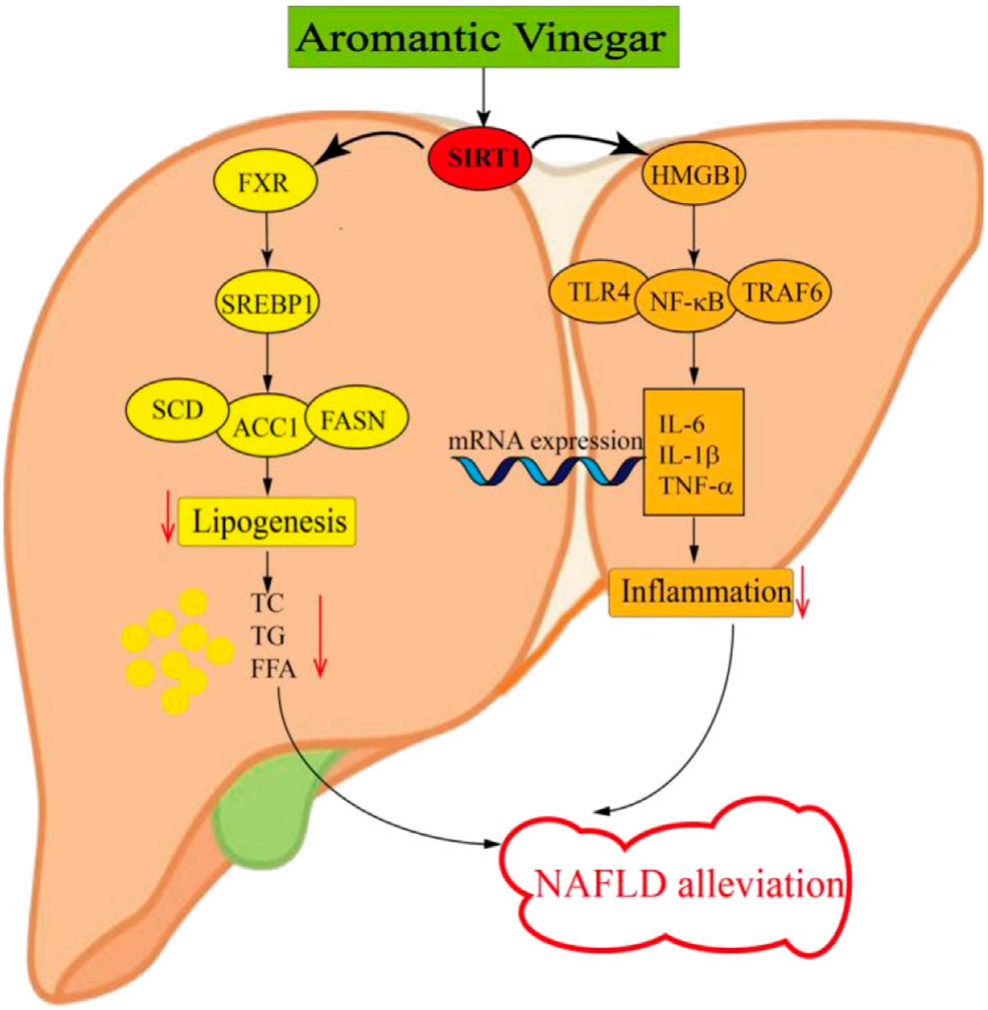
Schematic diagram of the anti-lipid metabolism and anti-inflammatory effect of Hengshun aromatic vinegar.

As a key regulator of hepatic metabolic homeostasis, hepatic SIRT1 acts as an endogenous activator of its downstream protein FXR in hepatocytes (Yang et al., 2017). FXR, a member of the nuclear receptor super family of ligand-activated transcription factors, has recently been demonstrated to play a key role in the molecular mechanism of regulating lipid glucose homeostasis (Nie et al., 2017). At the same time, FXR is the primary nuclear receptor for bile acids and plays an important role in bile acid homeostasis (Yang et al., 2017). In addition, FXR is a crucial target protein downstream of SIRT1, which plays a connecting role after being regulated by SIRT1. Generally speaking, hepatic FXR regulates lipid homeostasis through a variety of mechanisms, including reducing fatty acid synthesis, decreasing fatty acid uptake, and increasing *β*-oxidation (Cave et al., 2016). Celastrol has a protective effect on cholestatic liver injury by regulating SIRT1/FXR pathway (Zhao et al., 2019). Other research revealed that imperatorin exerts the protective effect against hepatotoxicity induced by excessive acetaminophen similarly by stimulating the SIRT1/FXR pathway (Gao et al., 2020). Moreover, studies have showed that NAFLD may be mitigated through the regulation of SIRT1/FXR signaling pathway (Han et al., 2019). Through the activation of FXR, a major adipogenic regulator called SREBP1 will be regulated and further reduced the protein levels of FASN, ACC1 and SCD in its downstream which are related to lipogenesis (Dong et al., 2019). Consistent with other research results, we found that FXR regulated by SIRT1 activates downstream protein SREBP1, which affects the protein levels of FASN, ACC1 and SCD, thereby reducing adipogenesis (Figure 8). Our results indicated that aromatic vinegar could alleviate NAFLD by reducing lipogenesis through SIRT1/FXR signaling pathway. What’s more, the SIRT1 siRNA experimental results also confirmed this conclusion.

It has been confirmed that SIRT1 not only regulates FXR, but also activates HMGB1, a key inflammatory mediator in the inflammatory signaling pathway (Qi et al., 2017). Recent study has demonstrated that SIRT1 is a key factor in the negative regulation of HMGB1, whose release is regulated by SIRT1 acetylation mediated direct interaction (Hwang et al., 2015). It has been reported that SIRT1 induces HMGB1 expression, thereby modulating ovarian cancer behaviors (Jiang et al., 2018). Le K et al. have demonstrated that resveratrol plays a neuroprotective role in neonatal hypoxic-ischemic brain injury by activating SIRT1 to inhibit HMGB1 signaling (Le et al., 2019). Moreover, The SIRT1/HMGB1 pathway has been proved to be an important target for inhibiting of NAFLD inflammation (Zeng et al., 2015). HMGB1, ubiquitously and highly expressed in hepatocytes, is a critical chromosomal non-histone protein that can regulate DNA structure. As we all know, it plays a pivotal role in the initiation and maintenance inflammation responses, which is a key process that takes place in ordinary liver diseases such as NAFLD (Khambu et al., 2019). The inflammation responses during NAFLD may be the result of stress, damage and cell death of hepatocytes caused by lipid, and such lipotoxicity further leads to the release of HMGB1 (Ibrahim et al., 2018). The downstream inflammation-related proteins such as TLR4, TRAF6 and NF-κB are activated due to the release of HMGB1 (Li et al., 2011; Lan et al., 2017). Researches have shown that liver inflammation is caused by pro-inflammatory cytokines and chemokines generated by adipocytes, lipid-laden hepatocytes and hepatic macrophages (Manne et al., 2018). NF-κB, activated by HMGB1, acts as a major transcriptional regulator regulating inflammation and cell death during the development of NAFLD (Jiang et al., 2019). After NF-κB activation, it is transferred from the cytoplasm to the nucleus, releasing a great deal of inflammatory factors. The alteration of NF-κB activity can affect the expression of hepatic pro-inflammatory cytokines, such as IL-1β, IL-6 and TNF-α, and the excessive production of which may lead to inflammatory reaction (Gäbele et al., 2009). In the present study, we obtained the following results that SIRT1 induced the release of HMGB1, which regulated the downstream inflammation-related proteins TLR4, TRAF6, and NF-κB. After activation, NF-κB released a large number of inflammatory factors including IL-1β, IL-6, and TNF-α (Figure 8). These results supported the view that aromatic vinegar played a critical role in relieving NAFLD by regulating inflammation through SIRT1/HMGB1 signaling pathway. In addition, the experimental results of SIRT1 siRNA also confirmed this conclusion.

There are some shortages in the current study. The anti-NAFLD effects of aromatic vinegar were evaluated only in cell and rats model, which needs to be further evaluated in clinical studies. It has been reported in Chinese journal that Hengshun aromatic vinegar concentrate, extract and its different molecular weight components could inhibit the oxidative modification of human low density lipoprotein. It provids the possibility for the clinical application of aromatic vinegar in treating NAFLD combined with our findings in this study. As flavoring, aromatic vinegar is difficult to be developed into a medicine for the treatment of diseases. Previous studies have suggested that osteoporosis was alleviated in rats by consumption of aromatic vinegar alone or in combination with calcium salt (Yu et al., 2020). Consequently, it will be a direction in the future that aromatic vinegar could be developed as a clinical adjunctive therapy to alleviate NAFLD and further studies could investigate the combination effect of Hengshun aromatic vinegar with other medicines.

## Conclusion

Our study demonstrated that Hengshun aromatic vinegar showed valid effects against NAFLD via regulating lipid metabolism and inflammation by targeting SIRT1. This study provided new evidence for the recommendation that Hengshun aromatic vinegar can be used as an adjuvant strategy for the therapy and health care of NAFLD.

## Data Availability Statement

The raw data supporting the conclusions of this article will be made available by the authors, without undue reservation, to any qualified researcher.

## Ethics Statement

The animal study was reviewed and approved by Animal Research Committee of Dalian Medical University.

## Author Contributions

Data curation, LX; Funding acquisition, GL; Methodology, SZ and LG; Project administration and resources, BZ; Software, MG; Validation, LG, XT, and CS; Writing – original draft, LG and LX; Writing – review and editing, LX. SZ and LG contributed same work to this paper and they are the co-first authors.

## Funding

This work was financially supported by the Key Research and Development Project of Zhenjiang, China (ZD2019001).

## Conflict of Interest

Authors SZ, GL, and BZ were employed by the company “Jiangsu Hengshun Vinegar Industry Co., Ltd.” (Zhenjiang, China).

The remaining authors declare that the research was conducted in the absence of any commercial or financial relationships that could be construed as a potential conflict of interest.

## References

[B1] Basaranoglu M. Basaranoglu G. Sentürk H. (2013). From fatty liver to fibrosis: a tale of “second hit”. World J. Gastroenterol 19, 1158–1165. 10.3748/wjg.v19.i8.1158 23483818 PMC3587471

[B2] Beh B. K. Mohamad N. E. Yeap S. K. Ky H. Boo S. Y. Chua J. Y. H. (2017). Anti-obesity and anti-inflammatory effects of synthetic acetic acid vinegar and Nipa vinegar on high-fat-diet-induced obese mice. Sci. Rep. 7, 6664 10.1038/s41598-017-06235-7 28751642 PMC5532206

[B3] Budak N. H. Aykin E. Seydim A. C. Greene A. K. Guzel-Seydim Z. B. (2014). Functional properties of vinegar. J. Food Sci. 79, R757–R764. 10.1111/1750-3841.12434 24811350

[B4] Bugianesi E. Moscatiello S. Ciaravella M. F. Marchesini G. (2010). Insulin resistance in nonalcoholic fatty liver disease. Curr. Pharm. Des. 16, 1941–1951. 10.2174/138161210791208875 20370677

[B5] Cave M. C. Clair H. B. Hardesty J. E. Falkner K. C. Feng W. Clark B. J. (2016). Nuclear receptors and nonalcoholic fatty liver disease. Biochim. Biophys. Acta 1859, 1083–1099. 10.1016/j.bbagrm.2016.03.002 26962021 PMC5149456

[B6] Chen B. Ma Y. Xue X. Wei J. Hu G. Lin Y. (2019). Tetramethylpyrazine reduces inflammation in the livers of mice fed a high fat diet. Mol. Med. Rep. 19, 2561–2568. 10.3892/mmr.2019.9928 30720104 PMC6423567

[B7] Chen H. Chen T. Giudici P. Chen F. (2016). Vinegar functions on health: Constituents, sources, and formation mechanisms. Compr. Rev. Food Sci. Food Saf. 15, 1124–1138. 10.1111/1541-4337.12228 33401833

[B8] Cheung O. Sanyal A. J. (2010). Recent advances in nonalcoholic fatty liver disease. Curr. Opin. Gastroenterol. 26, 202–208. 10.1097/MOG.0b013e328337b0c4 20168226

[B9] Chung K. W. Kim K. M. Choi Y. J. An H. J. Lee B. Kim D. H. (2017). The critical role played by endotoxin-induced liver autophagy in the maintenance of lipid metabolism during sepsis. Autophagy 13, 1113–1129. 10.1080/15548627.2017.1319040 28575583 PMC5529074

[B10] Colak Y. Ozturk O. Senates E. Tuncer I. Yorulmaz E. Adali G. (2011). SIRT1 as a potential therapeutic target for treatment of nonalcoholic fatty liver disease. Med. Sci. Mon. Int. Med. J. Exp. Clin. Res. 17, HY5 10.12659/msm.881749 PMC3539588 21525818

[B11] Colak Y. Yesil A. Mutlu H. H. Caklili O. T. Ulasoglu C. Senates E. (2014). A potential treatment of non-alcoholic fatty liver disease with SIRT1 activators. J. Gastrointestin. Liver Dis. 23, 311–319. 10.15403/jgld.2014.1121.233.yck 25267960

[B12] Dong R. Yang X. Wang C. Liu K. Liu Z. Ma X. (2019). Yangonin protects against non-alcoholic fatty liver disease through farnesoid X receptor. Phytomedicine 53, 134–142. 10.1016/j.phymed.2018.09.006 30668392

[B13] Dowman J. K. Armstrong M. J. Tomlinson J. W. Newsome P. N. (2011). Current therapeutic strategies in non-alcoholic fatty liver disease. Diabetes Obes. Metabol. 13, 692–702. 10.1111/j.1463-1326.2011.01403.x 21449949

[B14] Duan W. Xia T. Zhang B. Li S. Zhang C. Zhao C. (2019). Changes of physicochemical, bioactive compounds and antioxidant capacity during the brewing process of zhenjiang aromatic vinegar. Molecules 24, 3935 10.3390/molecules24213935 PMC6864686 31683587

[B15] Elvira-Torales L. I. Periago M. J. González-Barrio R. Hidalgo N. Navarro-González I. Gómez-Gallego C. (2019). Spinach consumption ameliorates the gut microbiota and dislipaemia in rats with diet-induced non-alcoholic fatty liver disease (NAFLD). Food Funct. 10, 2148–2160. 10.1039/c8fo01630e 30938723

[B16] Estes C. Razavi H. Loomba R. Younossi Z. Sanyal A. J. (2018). Modeling the epidemic of nonalcoholic fatty liver disease demonstrates an exponential increase in burden of disease. Hepatology 67, 123–133. 10.1002/hep.29466 28802062 PMC5767767

[B17] Farrell G. C. Larter C. Z. (2006). Nonalcoholic fatty liver disease: from steatosis to cirrhosis. Hepatology 43, S99–S112. 10.1002/hep.20973 16447287

[B18] Gäbele E. Froh M. Arteel G. E. Uesugi T. Hellerbrand C. Schölmerich J. (2009). TNFα is required for cholestasis-induced liver fibrosis in the mouse. Biochem. Biophys. Res. Commun. 378, 348–353. 10.1016/j.bbrc.2008.10.155 18996089 PMC5052129

[B19] Gao Z. Zhang J. Wei L. Yang X. Zhang Y. Cheng B. (2020). The protective effects of imperatorin on acetaminophen overdose-induced Acute liver injury. Oxidat. Med. Cell Longev. 2020, 1 10.1155/2020/8026838 PMC7243017 32454943

[B20] García-Alonso F. J. González-Barrio R. Martín-Pozuelo G. Hidalgo N. Navarro-González I. Masuero D. (2017). A study of the prebiotic-like effects of tomato juice consumption in rats with diet-induced non-alcoholic fatty liver disease (NAFLD). Food Funct. 8, 3542–3552. 10.1039/c7fo00393e 28876011

[B21] Gong Z. Tas E. Yakar S. Muzumdar R. (2017). Hepatic lipid metabolism and non-alcoholic fatty liver disease in aging. Mol. Cell. Endocrinol. 455, 115–130. 10.1016/j.mce.2016.12.022 28017785 PMC13080523

[B22] Han X. Cui Z.-Y. Song J. Piao H.-Q. Lian L.-H. Hou L.-S. (2019). Acanthoic acid modulates lipogenesis in nonalcoholic fatty liver disease via FXR/LXRs-dependent manner. Chem. Biol. Interact. 311, 108794 10.1016/j.cbi.2019.108794 31421115

[B23] Hwang J. S. Choi H. S. Ham S. A. Yoo T. Lee W. J. Paek K. S. (2015). Deacetylation-mediated interaction of SIRT1-HMGB1 improves survival in a mouse model of endotoxemia. Sci. Rep. 5, 15971 10.1038/srep15971 26522327 PMC4629154

[B24] Ibrahim S. H. Hirsova P. Gores G. J. (2018). Non-alcoholic steatohepatitis pathogenesis: sublethal hepatocyte injury as a driver of liver inflammation. Gut 67, 963–972. 10.1136/gutjnl-2017-315691 29367207 PMC5889737

[B25] Jiang J. Yan L. Shi Z. Wang L. Shan L. Efferth T. (2019). Hepatoprotective and anti-inflammatory effects of total flavonoids of Qu Zhi Ke (peel of Citrus changshan-huyou) on non-alcoholic fatty liver disease in rats via modulation of NF-κB and MAPKs. Phytomedicine 64, 153082 10.1016/j.phymed.2019.153082 31541796

[B26] Jiang W. Jiang P. Yang R. Liu D. F. (2018). Functional role of SIRT1-induced HMGB1 expression and acetylation in migration, invasion and angiogenesis of ovarian cancer. Eur. Rev. Med. Pharmacol. Sci. 22, 4431–4439. 10.26355/eurrev_201807_15494 30058682

[B27] Jing L. Yanyan Z. Junfeng F. (2015). Acetic acid in aged vinegar affects molecular targets for thrombus disease management. Food Funct. 6, 2845–2853. 10.1039/c5fo00327j 26189969

[B28] Khambu B. Yan S. Huda N. Yin X.-M. (2019). Role of high-mobility group box-1 in liver pathogenesis. Int. J. Mol. Sci. 20, 5314 10.3390/ijms20215314 PMC6862281 31731454

[B29] Kondo T. Kishi M. Fushimi T. Ugajin S. Kaga T. (2009). Vinegar intake reduces body weight, body fat mass, and serum triglyceride levels in obese Japanese subjects. Biosci. Biotechnol. Biochem. 73, 1837–1843. 10.1271/bbb.90231 19661687

[B30] Lan K.-C. Chao S.-C. Wu H.-Y. Chiang C.-L. Wang C.-C. Liu S.-H. (2017). Salidroside ameliorates sepsis-induced acute lung injury and mortality via downregulating NF-κB and HMGB1 pathways through the upregulation of SIRT1. Sci. Rep. 7, 12026 10.1038/s41598-017-12285-8 28931916 PMC5607272

[B31] Le K. Chibaatar Daliv E. Wu S. Qian F. Ali A. I. Yu D. (2019). SIRT1-regulated HMGB1 release is partially involved in TLR4 signal transduction: a possible anti-neuroinflammatory mechanism of resveratrol in neonatal hypoxic-ischemic brain injury. Int. Immunopharm. 75, 105779 10.1016/j.intimp.2019.105779 31362164

[B32] Li L. Chen L. Hu L. Liu Y. Sun H.-Y. Tang J. (2011). Nuclear factor high-mobility group box1 mediating the activation of toll-like receptor 4 signaling in hepatocytes in the early stage of nonalcoholic fatty liver disease in mice. Hepatology 54, 1620–1630. 10.1002/hep.24552 21809356

[B33] Manne V. Handa P. Kowdley K. V. (2018). Pathophysiology of nonalcoholic fatty liver disease/nonalcoholic steatohepatitis. Clin. Liver Dis. 22, 23–37. 10.1016/j.cld.2017.08.007 29128059

[B34] Nie H. Song C. Wang D. Cui S. Ren T. Cao Z. (2017). MicroRNA-194 inhibition improves dietary-induced non-alcoholic fatty liver disease in mice through targeting on FXR. Biochim. Biophys. Acta 1863, 3087–3094. 10.1016/j.bbadis.2017.09.020 28951211

[B35] Qi Z. Zhang Y. Qi S. Ling L. Gui L. Yan L. (2017). Salidroside inhibits HMGB1 acetylation and release through upregulation of SirT1 during inflammation. Oxidat. Med. Cell Longev. 2017, 1 10.1155/2017/9821543 PMC5733170 29333216

[B36] Qin G. Ma J. Huang Q. Yin H. Han J. Li M. (2018). Isoquercetin improves hepatic lipid accumulation by activating AMPK pathway and suppressing TGF-β signaling on an HFD-induced nonalcoholic fatty liver disease rat model. Int. J. Mol. Sci. 19, 4126 10.3390/ijms19124126 PMC6321444 30572631

[B37] Rolo A. P. Teodoro J. S. Palmeira C. M. (2012). Role of oxidative stress in the pathogenesis of nonalcoholic steatohepatitis. Free Radic. Biol. Med. 52, 59–69. 10.1016/j.freeradbiomed.2011.10.003 22064361

[B38] Safari Z. Gérard P. (2019). The links between the gut microbiome and non-alcoholic fatty liver disease (NAFLD). Cell. Mol. Life Sci. 76, 1541–1558. 10.1007/s00018-019-03011-w 30683985 PMC11105223

[B39] Shen F. Feng J. Wang X. Qi Z. Shi X. An Y. (2016). Vinegar treatment prevents the development of murine experimental colitis via inhibition of inflammation and apoptosis. J. Agric. Food Chem. 64, 1111–1121. 10.1021/acs.jafc.5b05415 26795553

[B40] Silva J. P. Wahlestedt C. (2010). Role of Sirtuin 1 in metabolic regulation. Drug Discov. Today 15, 781–791. 10.1016/j.drudis.2010.07.001 20621197

[B41] Soares e Silva A. K. de Oliveira Cipriano Torres D. dos Santos Gomes F. O. dos Santos Silva B. Lima Ribeiro E. Costa Oliveira A. (2015). LPSF/GQ-02 inhibits the development of hepatic steatosis and inflammation in a mouse model of non-alcoholic fatty liver disease (NAFLD). PLoS One 10, e0123787 10.1371/journal.pone.0123787 25875942 PMC4397012

[B42] Stefan N. Häring H.-U. Cusi K. (2019). Non-alcoholic fatty liver disease: causes, diagnosis, cardiometabolic consequences, and treatment strategies. Lancet Diabetes Endocrinol. 7, 313–324. 10.1016/S2213-8587(18)30154-2 30174213

[B43] Tang W. Yao X. Xia F. Yang M. Chen Z. Zhou B. (2018). Modulation of the gut microbiota in rats by hugan qingzhi tablets during the treatment of high-fat-diet-induced nonalcoholic fatty liver disease. Oxidat. Med. Cell Longev. 2018, 1 10.1155/2018/7261619 PMC6323444 30671174

[B44] Wu X. Xu N. Li M. Huang Q. Wu J. Gan Y. (2019). Protective effect of patchouli alcohol against high-fat diet induced hepatic steatosis by alleviating endoplasmic reticulum stress and regulating VLDL metabolism in rats. Front. Pharmacol. 10, 1134 10.3389/fphar.2019.01134 31632274 PMC6779828

[B45] Xia H. Zhu X. Zhang X. Jiang H. Li B. Wang Z. (2019). Alpha-naphthoflavone attenuates non-alcoholic fatty liver disease in oleic acid-treated HepG2 hepatocytes and in high fat diet-fed mice. Biomed. Pharmacother. 118, 109287 10.1016/j.biopha.2019.109287 31401392

[B46] Xu W. Huang Z. Zhang X. Li Q. Lu Z. Shi J. (2011a). Monitoring the microbial community during solid-state acetic acid fermentation of Zhenjiang aromatic vinegar. Food Microbiol. 28, 1175–1181. 10.1016/j.fm.2011.03.011 21645817

[B47] Xu W. Xu Q. Chen J. Lu Z. Xia R. Li G. (2011b). Ligustrazine formation in Zhenjiang aromatic vinegar: changes during fermentation and storing process. J. Sci. Food Agric. 91, 1612–1617. 10.1002/jsfa.4356 21452171

[B48] Yang J. Sun L. Wang L. Hassan H. M. Wang X. Hylemon P. B. (2017). Activation of SIRT1/FXR signaling pathway attenuates triptolide-induced hepatotoxicity in rats. Front. Pharmacol. 8, 260 10.3389/fphar.2017.00260 28536529 PMC5422577

[B49] Yao H. Tao X. Xu L. Qi Y. Yin L. Han X. (2018). Dioscin alleviates non-alcoholic fatty liver disease through adjusting lipid metabolism via SIRT1/AMPK signaling pathway. Pharmacol. Res. 131, 51–60. 10.1016/j.phrs.2018.03.017 29574225

[B50] Younossi Z. M. Koenig A. B. Abdelatif D. Fazel Y. Henry L. Wymer M. (2016). Global epidemiology of nonalcoholic fatty liver disease-meta-analytic assessment of prevalence, incidence, and outcomes. Hepatology 64, 73–84. 10.1002/hep.28431 26707365

[B51] Yu Y. Zhang Z. Zhang B. Zeng Y. Li X. Ji H. (2020). Implication of vinegar alone or in combination with caltrate in a rat osteoporosis model induced by a low calcium diet and retinoic acid. Chin. Tradit. Med. J. 3, 1–8.

[B52] Zeng W. Shan W. Gao L. Gao D. Hu Y. Wang G. (2015). Inhibition of HMGB1 release via salvianolic acid B-mediated SIRT1 up-regulation protects rats against non-alcoholic fatty liver disease. Sci. Rep. 5, 16013 10.1038/srep16013 26525891 PMC4630617

[B53] Zhang B. Xia T. Duan W. Zhang Z. Li Y. Fang B. (2019). Effects of organic acids, amino acids and phenolic compounds on antioxidant characteristic of Zhenjiang aromatic vinegar. Molecules 24, 3799 10.3390/molecules24203799 PMC6832349 31652563

[B54] Zhang X. Ji X. Wang Q. Li J. Z. (2018). New insight into inter-organ crosstalk contributing to the pathogenesis of non-alcoholic fatty liver disease (NAFLD). Protein Cell 9, 164–177. 10.1007/s13238-017-0436-0 28643267 PMC5818366

[B55] Zhang Y. Geng C. Liu X. Li M. Gao M. Liu X. (2017). Celastrol ameliorates liver metabolic damage caused by a high-fat diet through SIRT1. Mol. Metabol. 6, 138–147. 10.1016/j.molmet.2016.11.002 PMC5220393 28123944

[B56] Zhao C. Xia T. Du P. Duan W. Zhang B. Zhang J. 2018). Chemical composition and antioxidant characteristic of traditional and industrial Zhenjiang aromatic vinegars during the aging process. Molecules 23, 2949 10.3390/molecules23112949 PMC6278357 30424522

[B57] Zhao Q. Liu F. Cheng Y. Xiao X.-R. Hu D.-D. Tang Y.-M. (2019). Celastrol protects from cholestatic liver injury through modulation of SIRT1-FXR signaling. Mol. Cell. Proteomics 18, 520–533. 10.1074/mcp.RA118.000817 30617157 PMC6398203

[B58] Zhu W. Liu Y. Lan Y. Li X. Luo L. Duan X. (2019). Dietary vinegar prevents kidney stone recurrence via epigenetic regulations. EBioMedicine 45, 231–250. 10.1016/j.ebiom.2019.06.004 31202812 PMC6642359

